# Protective Effects of Evening Primrose Oil against Cyclophosphamide-Induced Biochemical, Histopathological, and Genotoxic Alterations in Mice

**DOI:** 10.3390/pathogens9020098

**Published:** 2020-02-05

**Authors:** Dina M. Khodeer, Eman T. Mehanna, Abdelrahman I. Abushouk, Mohamed M. Abdel-Daim

**Affiliations:** 1 Department of Pharmacology and Toxicology, Faculty of Pharmacy, Suez Canal University, Ismailia 41522, Egypt; dina_khoudaer@pharm.suez.edu.eg; 2 Department of Biochemistry, Faculty of Pharmacy, Suez Canal University, Ismailia 41522, Egypt; eman.taha@pharm.suez.edu.eg; 3 Faculty of Medicine, Ain Shams University, Cairo 11566, Egypt; 4 Department of Medicine, Harvard Medical School, Boston, MA 02215, USA; 5 Department of Zoology, College of Science, King Saud University, Riyadh 11451, Saudi Arabia; abdeldaim.m@vet.suez.edu.eg; 6 Pharmacology Department, Faculty of Veterinary Medicine, Suez Canal University, Ismailia 41522, Egypt

**Keywords:** cyclophosphamide, evening primrose oil, insulin, liver, pancreas, mice

## Abstract

Cyclophosphamide (CP) is a well-known antineoplastic agent; however, its clinical use can be associated with various organ toxicities. Evening primrose oil (EPO) contains several phytoconstituents with potent anti-oxidant and anti-inflammatory activities. This experimental study was performed to investigate the chemoprotective effects of EPO in the liver and pancreas of CP-intoxicated mice. Thirty-two albino mice were randomly divided into 4 equal groups: group I received saline (control mice), group II were treated with CP at 100 mg/kg/day for two subsequent days, and groups III and VI were treated with 5 and 10 mg/kg/day bw EPO, respectively for 14 days, followed by two doses of CP at the 15th and 16th days of the experiment. Then, mice were sacrificed and histopathological examinations, biochemical studies, and DNA laddering tests were conducted for hepatic and pancreatic tissues. Cyclophosphamide-intoxicated mice showed significant increases (*p* < 0.05) in the serum levels of liver enzymes, pancreatic amylase and tissue levels of malondialdehyde, and TNF-α, as well as a significant decrease (*p* < 0.05) in the serum insulin level. In addition, both hepatic and pancreatic tissues showed disturbed tissue architecture, hydropic degeneration, congested vessels, and inflammatory infiltrates, as well as increased DNA fragmentation. In a dose-dependent manner, pretreatment with EPO was associated with significant improvements (*p* < 0.05) in all biochemical parameters and significant amelioration of histopathological alterations and DNA fragmentation in CP-intoxicated mice. Pretreatment with EPO showed significant antioxidant, anti-inflammatory, and genoprotective effects against the toxic effects of CP in mice hepatic and pancreatic tissues.

## 1. Introduction

Cyclophosphamide (CP) is an alkylating chemotherapeutic drug that belongs to the class of oxazaphosporines [1,2]. It is widely used to treat various types of cancer, including lymphoma, leukemia, breast, ovarian, and lung carcinomas [3], as well as autoimmune diseases as rheumatoid arthritis (due to its immunosuppressive effects) [1]. Cyclophosphamide is metabolized via the cytochrome P450 system, producing acrolein and phosphoramide mustard [4]. Acrolein is a reactive aldehyde that generates toxic reactive oxygen species (ROS), leading to oxidative stress [5]. Cyclophosphamide toxicity affects many organs, including the liver and pancreas, and is characterized by depletion of glutathione, lipid peroxidation, altered DNA profile, pro-inflammatory response, and apoptosis [6,7]. 

Evening primrose oil (EPO) [*Oenothera biennis*] is a well-known alternative medication, with diverse phytoconstituents including esters, alcohols, triterpenoids, fatty acids, phenolic acids, lactones, flavonoids, tannins, sterols, and chalcone [8]. It is used traditionally as an antidiabetic drug and to treat inflammatory diseases as atopic dermatitis and rheumatoid arthritis [9]. Several studies confirmed the radical-scavenging and anti-oxidant effects of EPO [8,10]. Further, it was found to exert a strong lipoxygenase inhibitory effect, accounting for its anti-inflammatory activity [11]. Other research groups highlighted its hypoglycemic, hypocholesterolemic [12], anti-bacterial, and anti-fungal [13] properties. Evening primrose oil supplements, rich in linoleic and linolenic acids also improved neural function in breast cancer patients suffering from chemotherapy-induced neuropathy [14].

**Figure 1:**
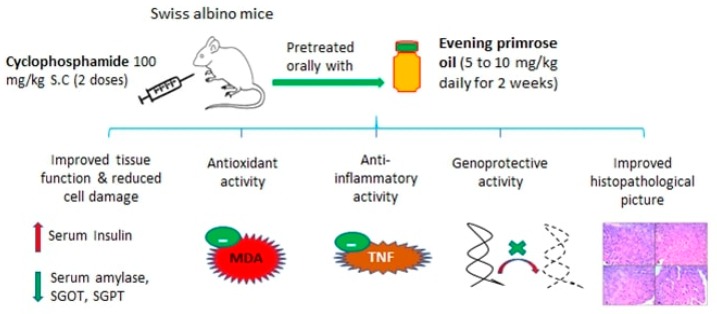
Summary of the experimental design and findings.

To our knowledge, there are no published experiments in the literature over the potential chemoprotective effects of EPO against the cytotoxicity of chemotherapeutics. Therefore, the current study was aimed to investigate the protective effects of EPO against CP-induced biochemical, histopathological, and genotoxic alterations in mice hepatic and pancreatic tissues (Figure 1).

## 2. Results

### 2.1. GC-MS Analysis Results 

**Figure 2:**
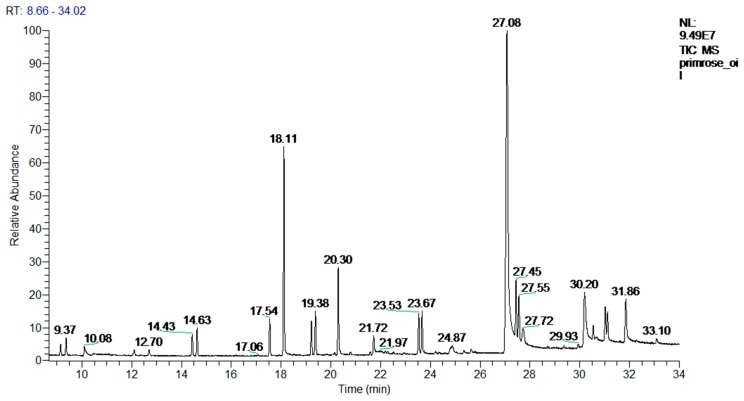
Total ion chromatogram obtained by GC–MS analysis of evening primrose oil.

Twenty compounds were identified in the used EPO sample. The major components in the analyzed samples were 3β-Sitosterol [stigmast-5-en-3-ol, (3β)-; 39.8%], campesterol [ergost-5-en-3-ol (3β24R); 17.1%], caryophyllene [8.2%], and 2-(1,1-dimethylethyl)-5-(2-propenyl)-1,4-benzenediol [5.4%]. The retention time for these compounds ranged from 8.66 to 34.02 min. The total ion chromatogram obtained by GC–MS analysis of primrose oil is illustrated in Figure 2 and the full list of present compounds and their peak areas are shown in Table 1.

### 2.2. Serum Biochemical Analysis

Compared to control mice, CP-intoxicated mice had significantly higher (*p* < 0.05) serum concentrations of serum glutamic pyruvic transaminase (SGPT), serum glutamic oxaloacetic transaminase (SGOT), and pancreatic amylase, as well as significantly lower (*p* < 0.05) serum concentrations of insulin. However, mice pretreated with EPO at 5 or 10 mg/kg/day showed significant amelioration of these changes. Pretreatment with EPO at 10 mg/kg/day restored the control ranges of liver enzymes, pancreatic amylase, and insulin (Table 2).

### 2.3. Tissue Biochemical Analysis

Significant increases (*p* < 0.05) were recorded in hepatic and pancreatic tissue concentrations of malondialdehyde (MDA) and tumor necrosis factor alpha (TNF-α) following CP injections, in comparison to control mice. However, pretreatment with either dose of EPO (5 or 10 mg/kg/day) alleviated these increases. The hepatic concentrations of MDA and TNF-α in mice pretreated with the 10 mg/kg/day dose of EPO were significantly lower (*p* < 0.05) than in those pretreated with the 5 mg/kg/day dose; Table 3.

### 2.4. Histopathological Examination and Analysis

**Figure 3:**
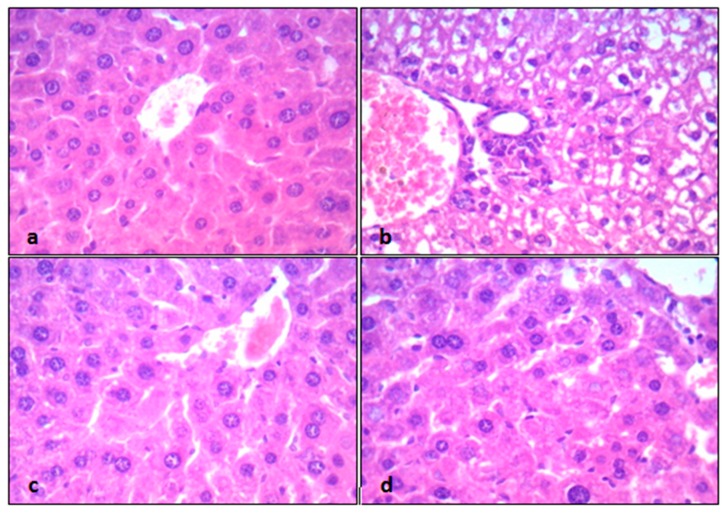
The protective effect of different doses of EPO on hepatic histopathology, induced by cyclophosphamide: (**a**) Control mice, (**b**) CP-intoxicated mice, (**c**) CP + EPO 5 mg/kg/day mice, and (**d**) CP + EPO 10 mg/kg/day mice. All sections captured at 400× magnification, using objective 40×, UIS optical system (Universal Infinity System, Olympus^®^, Tokyo, Japan).

Liver tissue sections from control mice showed preserved tissue architecture, formed of hepatocytes with abundant cytoplasm and small nuclei, arranged in thin cell trabeculae, and separated by thin-wall blood sinusoids with normal central vein and portal tracts (Figure 3a). In contrast, CP-intoxicated mice showed disturbed tissue architecture; hepatocytes arranged in thick and thin cell trabeculae with marked hydropic degeneration, congested dilated central veins, compressed sinusoids (due to hydropic degeneration and cell swelling of hepatocytes), and mild inflammatory infiltrates (Figure 3b). However, group III mice (CP + EPO 5 mg/kg/day) showed preserved hepatic tissue architecture; hepatocytes arranged in thin cell trabeculae with mild residual hydropic degeneration and normal sinusoids with slightly congested vessels (Figure 3c). Group IV mice (CP + EPO 10 mg/kg/day) showed marked improvements with hepatocytes arranged in thin cell trabeculae in a lobular architecture, separated by thin-wall blood sinusoids (Figure 3d). 

**Figure 4:**
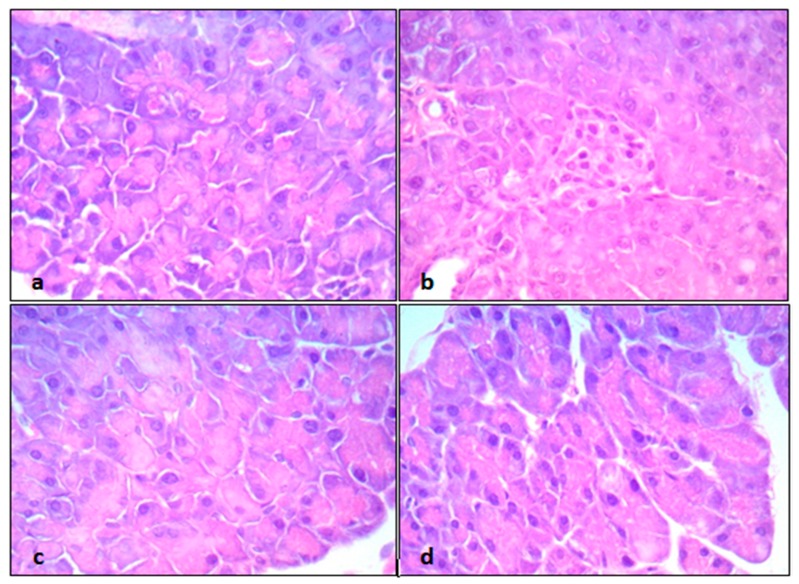
The protective effect of different doses of EPO on pancreatic histopathology, induced by cyclophosphamide: (**a**) Control mice, (**b**) CP-intoxicated mice, (**c**) CP + EPO 5 mg/kg/day mice, and (**d**) CP + EPO 10 mg/kg/day mice. All sections captured at 400× magnification, using objective 40×, UIS optical system (Universal Infinity System, Olympus^®^, Tokyo, Japan).

Similarly, pancreatic tissue sections from control mice showed normal pancreatic acini with basal nuclei and amphophilic cytoplasm. The cells of the islets of Langerhans showed abundant eosinophilic cytoplasm and central small nuclei, and were arranged in trabecular and acinar patterns (Figure 4a). However, the pancreatic acini in CP-intoxicated mice showed focal moderate hydropic degeneration and moderate edema and congestion. In addition, the islets of Langerhans were markedly irregular and reduced in size with atrophic and shrunken cells (Figure 4b). On the other hand, group III mice (CP + EPO 5 mg/kg/day) showed some improvement with a residual decrease in the size of islets of Langerhans, mild degeneration of pancreatic acini, and moderate congestion (Figure 4c). Group IV (CP + EPO 10 mg/kg/day) mice showed marked improvements with normal pancreatic acini and regular, normal-sized islets of Langerhans with abundant eosinophilic cytoplasm and central small nuclei (Figure 4d). Histopathological scoring confirmed the significant protective effects of primrose oil against CP-induced hepatic and pancreatic damages (Table 4).

### 2.5. DNA Laddering

**Figure 5:**
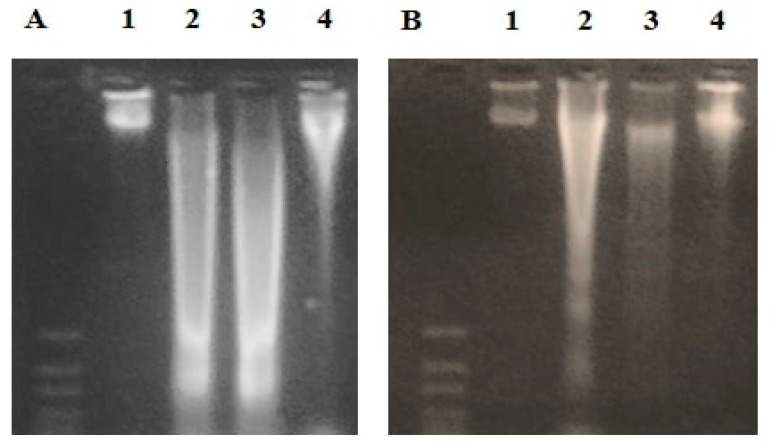
Horizontal gel electrophoresis of genomic DNA extracted from (**A**) liver, and (**B**) pancreas of experimental mice. Lane (**1**): Control mice. Lane (**2**): CP-intoxicated mice. Lane (**3**): CP + EPO (5 mg/kg/day). Lane (**4**): CP + EPO (10 mg/kg/day).

DNA laddering assay (Figure 5) showed that in both hepatic (A) and pancreatic (B) tissues, CP administration (lane 2) caused remarkable fragmentation of genomic DNA compared to control mice (lane 1). In the group treated with EPO (5 mg/kg/day) prior to CP administration, DNA fragmentation was slightly decreased (lane 3). However, there was a marked reduction of DNA damage in the group treated with EPO at 10 mg/kg/day (lane 4), especially in the pancreatic tissue.

## 3. Discussion

In the current study, acute intoxication with CP caused significant injuries to mice hepatic and pancreatic tissues as revealed by biochemical, histopathological, and genotoxicity analyses. Cyclophosphamide is an anti-neoplastic agent, incorporated in several anti-cancer regimens. There is evidence in the literature for CP-induced hepatotoxicity [15,16] and some reports of CP-related pancreatic dysfunction and diabetic changes [17,18]. The suggested mechanisms for CP-induced liver toxicity include exposure to the CP metabolite (*o*-carboxyethyl-phosphoramide mustard), which is related to biliary obstruction [15], oxidative stress (related to another metabolite: acrolein) [5], and lymphocytic infiltration [19]. The CP-induced pancreatic toxicity may be an idiosyncratic reaction or secondary to hepatotoxicity [18]. 

Cyclophosphamide acts partly through increasing the production of ROS in malignant cells; however, it also induces oxidative stress in different body tissues [5]. In our study, subcutaneous (S.C) injection with two subsequent doses of CP was associated with significant increases in hepatic and pancreatic MDA concentrations. Similar findings were reported in earlier investigations [20,21]. This reflects increased lipid peroxidation in CP-intoxicated mice. The CP-induced oxidative stress may also explain the observed genotoxicity, manifested as increased levels of DNA fragmentation in our DNA laddering experiment. 

In addition, intoxication with CP was associated with significant increases in both hepatic and pancreatic tissue concentrations of TNF-α. Ohtani et al. [22] showed that 4-hydroxycyclophosphamide (4-HC), a metabolite of CP enhances TNF-α mediated DNA fragmentation (which was also observed in the current study). Further, TNF-α was shown to play a role in CP-induced apoptosis in vascular endothelial cells [22] and hemorrhagic cystitis [23]. Our findings uncover the involvement of TNF-α in CP-induced toxicity in other body tissues (liver and pancreas).

Histopathological examination of liver tissue sections in CP-intoxicated mice showed disturbed tissue architecture, hydropic degeneration, congested central veins, and inflammatory infiltrates. Similar findings were noticed in CP-intoxicated mice after a single intraperitoneal dose of 200 mg/kg of CP [19]. In the pancreatic tissue, CP treatment was associated with atrophic islets of *Langerhans* with hydropic degeneration and moderate edema. In the same vein, former studies showed that CP can induce accelerated diabetes in mice via caspase-3 overexpression and B-cell apoptosis [24,25].

Interestingly, pretreatment with EPO (at two doses: 5 and 10 mg/kg/day for 14 days) before CP intoxication ameliorated all investigated CP-induced oxidative, inflammatory, histopathological, and genotoxic changes. Previous studies have highlighted the antioxidant potential of EPO in vitro [26] and in vivo in animal models with subacute aflatoxin toxicity [27] and hyperlipidemia [12]. Further, EPO treatment reduced the DNA fragmentation, induced by CP. Similarly, a former study showed that EPO can ameliorate the genotoxicity of ifosfamide [28]. These effects may be mediated by the established antioxidant effects of EPO, which were further confirmed in this study by the significant reduction of tissue MDA in EPO-treated mice. Of note, CP systemic toxicity is related to hepatic CP metabolism that produces toxic metabolites. Therefore, EPO may ameliorate CP toxicity by reducing the generation of CP toxic metabolites in the liver. However, this mechanism needs further confirmation. 

In addition, EPO showed marked anti-inflammatory effects in our study as manifested by the significant reduction of tissue TNF-α levels and amelioration of inflammatory infiltrates on histopathological examination in mice, pretreated with EPO. The anti-inflammatory effects of EPO were previously reported in vitro [26], and in clinical studies in diabetes [10], rheumatoid arthritis [29], and atopic dermatitis [30]. One suggested mechanism was that EPO is rich in dihomo-γ-linolenic acid that stops the transformation of arachidonic acid into leukotrienes [29]. The observed effects of EPO on TNF-α in our study confirm the findings of prior studies in the literature [31,32].

Another interesting finding in our study was the increased insulin secretion in EPO-treated mice and restoration of normal insulin levels in the 10 mg/kg bw EPO group. Published data shows that EPO can increase insulin secretion [33], ameliorate metabolic abnormalities in diabetic patients [12] and reduce the risk of vascular complications [10,34]. Improved insulin secretion may be attributed to the reduction of oxidative stress, inflammation, and subsequent destruction of β-cells (as demonstrated in our histopathological examination).

To identify the active constituents in our sample that may be responsible for the observed protective effects, we performed GC-MS analysis. The major component in our sample was 3β-Sitosterol. This compound has been shown to possess antioxidant, anti-inflammatory, and antidiabetic effects [35,36] and may be responsible in part for the observed effects for evening primrose oil. Similar effects were reported for β-Caryophyllene [37]. Another major component was Campesterol, which -along with 3β-Sitosterol were shown before to modulate the release of pro-inflammatory cytokines [32]. However, other less abundant molecules may be responsible for these effects as abundance does not equal efficacy; thus, future studies should determine the exact molecules to which the observed benefits of EPO can be attributed. Further, to confirm that EPO does not antagonize the anti-tumor activity of CP, further comparative studies in tumor-bearing mice are planned. Therefore, more data are needed before translating the findings of the current study into human research. 

## 4. Materials and Methods 

### 4.1. Ethics Statement

All used experimental procedures were approved by the Research Ethics Committee at the Faculty of Pharmacy, Suez Canal University, Ismailia, Egypt (Approval No. 201512A9).

### 4.2. Experimental Animals

Male Swiss albino mice (weighing 28–35 g) were supplied by the Egyptian Organization for Biological Products and Vaccines (Cairo, Egypt). Mice were housed in groups of eight in polyethylene cages with normal dark/light cycle and temperature between 25 ± 3 °C. Mice could acclimatize for 7 days before initiating the experiment and were freely provided with water and food *ad libitum*.

Cyclophosphamide monohydrate (CAS No: 6055-19-2) was obtained from Sigma-Aldrich (St. Louis, MO, USA), then prepared by dissolving in water (5 g/100 mL at 23 °C) taking in consideration that the aqueous solution of cyclophosphamide is light-sensitive. Evening primrose oil (*Primrose* Plus^®^ capsules) was purchased from EMA-Pharmaceuticals (Moncay, Lailly-en-Val, France). 

To assess EPO chemical composition, we used Trace GC Ultra-ISQ mass spectrometer (Thermo Scientific, Austin, TX, USA). For a start, we held the column oven temperature at 60 °C and then increased it to 220 °C (held for 2 min), and then to 300 °C (at a rate of 5 °C/min). The MS transfer line and injector temperatures were kept at 270 °C. Using Autosampler AS3000 along with GC (split mode), we injected diluted samples of 1 µL (with a solvent delay of 3 min). We collected EI mass spectra at 70 eV ionization voltages over the range of *m*/*z* 40–650 in full-scan mode. The transfer line and ion source were set at 280 °C and 200 °C, respectively. The sample components were identified by comparing their mass spectra and retention times with those of the WILEY 09/NIST 11 database.

### 4.3. Experimental Design

Mice were randomly divided into four equal groups. Group I mice were treated with saline (control mice); group II mice were treated with two S.C doses of CP (100 mg/kg/day on the 15th and 16th days of the experiment = 200 mg/kg total) [2]; while group III and group VI mice were treated with 5 and 10 mg/kg/day bw EPO respectively for 14 days [28] followed by two S.C doses of CP (100 mg/kg/day) on the 15th and 16th day of the experiment. 

### 4.4. Sample Collection and Preparation

Mice were sacrificed under isoflurane anesthesia. The liver and pancreas of each mouse were rapidly dissected and washed with ice cold NaCl 0.9% solution. Parts of the liver (0.3 g) and pancreas (0.1 g) were blotted and kept at −80 °C. These tissues were homogenized in phosphate buffer (pH 7.4) for biochemical assays, and then centrifuged at 3000× *g* and 4 °C for 15 min. 

### 4.5. Measurement of Biochemical Parameters

#### 4.5.1. Determination of Serum Level of Liver Enzymes and Pancreatic Amylase 

Blood samples were withdrawn from each rat from the orbital sinus to obtain the sera which were used to determine the concentrations of SGPT and SGOT using commercial kits (Biocon Diagnostic, Vöhl, Germany) and a UV-visible spectrophotometer (UV-1601PC, Shimadzu, Japan). The serum α-amylase activity was determined by an enzymatic colorimetric test using 2-choloro-4-nitrofenylo-α-maltrioside (CNPG3) [38].

#### 4.5.2. Determination of Fasting Serum Insulin Level

Fasting serum insulin was estimated using an enzyme-linked immunosorbent assay (ELISA) kit for insulin (Biorbyt, UK) following the manufacturer’s protocols (Crystal Chem Inc., Downers Grove, IL, USA).

#### 4.5.3. Measurement of Tissue Homogenate Level of TNF-α and MDA

The tissue concentrations of TNF-α were determined, using a commercially available ELISA Kit (Biosource^®^, Camarillo, CA, USA) following the instructions of the manufacturer. TNF-α was expressed as pg/g tissue. The tissue MDA concentration was measured following the methods of Mihara and Uchiyama [39].

### 4.6. Histopathological Examination

Additionally, liver and pancreas tissue samples were cut 5 mm apart from the edge of the largest hepatic lobe and the remaining part of the pancreas, respectively, and then fixed with 10% (*v*/*v*) formaldehyde and paraffin wax, and stained with hematoxylin and eosin (H&E) stain for histopathological examinations. An experienced pathologist who was masked to the animal groups conducted the histological examinations. A semiquantitative method, proposed by Dixon et al. [40], was used to assess the observed pathological changes.

### 4.7. DNA Laddering

Genomic DNA was extracted and purified form homogenized liver and pancreas tissues using Wizard^®^ Genomic DNA Purification kit (Promega, Madison, WI, USA). Extracted DNA was tested for concentration and purity by Nanodrop^®^ NA-1000 UV/Vis (ThermoFisher spectrophotometer, Wilmington, DE, USA). DNA fragmentation was assessed by horizontal electrophoresis using 2% agarose gel stained with ethidium bromide. A total of 10 μL of each DNA sample was mixed with 2 μL of the loading dye (ThermoFischer Scientific Inc, Waltham, MA, USA). Mixed samples were loaded to the prepared agarose gel. Electric current (90 volt) was applied for 45 min followed by visualization using UV trans-illuminator.

### 4.8. Statistical Analysis

Results were expressed as mean ± standard error of mean (SEM) and analyzed using the SPSS program version 16 (Chicago, IL, USA). Numerical data were compared using the one-way analysis of variance, supplemented by Bonferroni’s multiple comparisons test. Differences were considered significant at *p* ≤ 0.05.

## 5. Conclusions

Pretreatment with EPO—in a dose-dependent manner—showed significant antioxidant, anti-inflammatory, and genoprotective effects against the toxic effects of CP. This resulted in marked alleviation of hepatic and pancreatic tissue injuries in CP-intoxicated mice.

## Figures and Tables

**Table 1:** The chemical composition of evening primrose oil by GC-MS analysis.

Peak	Retention Time	Name	Area%	Molecular Weight	Molecular Formula
1	4.08	Decane	1.39	142	C10H22
2	6.63	1-Hexadecanol, 2-methyl-	1.13	256	C17H36O
3	9.36	Dodecane	1.62	170	C12H26
4	10.10	Thiophene, tetrahydro-, 1,1-dioxide	1.52	120	C4H8O2S
5	12.10	2,4-Dodecadienal, (E,E)-	0.88	180	C12H20O
6	12.70	Deca-2,4-Dienal	1.25	152	C10H16O
7	14.43	1-Dodecene	0.77	168	C12H24
8	14.63	(3β,5α)-Cholestanol	2.52	389	C27H48O
9	17.54	2-(1,1-dimethylethyl)-5-(2-propenyl)-1,4-benzenediol	5.38	206	C13H18O2
10	18.11	Ergost-5-en-3-ol(3β24R)	17.10	401	C28H48O
11	19.22	7-Hexadecene, (Z)-	1.24	224	C16H32
12	19.38	Hexadecane	1.63	226	C16H34
13	20.30	Caryophyllene	8.23	204	C15H24
14	21.73	3-Nonanol, 2-methyl-	0.77	158	C10H22O
15	23.67	Nonadecane	1.63	268	C19H40
16	27.08	Stigmast-5-en-3-ol, (3β)-	39.81	415	C29H50O
17	27.44	1-Eicosanol	2.05	298	C20H42O
18	27.73	Gibberellic acid	3.92	346	C19H22O6
19	30.20	Linoleic acid ethyl ester	4.51	308	C20H36O2
20	31.85	8,11,14-Eicosatrienoic acid, (Z,Z,Z)-	2.12	306	C20H34O2
			Σ99.47		

**Table 2:** The protective effects of evening primrose oil against cyclophosphamide-induced changes in serum levels of liver enzymes (SGOT and SGPT), pancreatic amylase, and insulin.

Groups	SGPT u/L	SGOT u/L	Amylase u/L	Insulin ng/mL
Control	9.87 ± 0.44	35.6 ± 3.22	29.7 ± 1.2	10.43 ± 0.3
CP-intoxicated	24.6 ± 1.7 ^a^	68.6 ± 1.7 ^a^	55.2 ± 1.8 ^a^	5.5 ± 0.26 ^a^
CP + EPO (5 mg/kg)	14.6 ± 1.26 ^ab^	49.12 ± 1.96 ^ab^	44.1 ± 1.98 ^ab^	7.1 ± 0.22 ^ab^
CP + EPO (10 mg/kg)	11.87 ± 0.6 ^b^	41.1 ± 0.55 ^bc^	33.6 ± 2.7 ^bc^	10.7 ± 0.69 ^bc^

All data are expressed as mean ± SEM and analyzed using the one-way ANOVA, followed by Bonferroni’s post-hoc test at *p* ˂ 0.05. ^a^ Significantly different from the control mice, ^b^ Significantly different from CP-intoxicated mice, ^c^ Significantly different from CP + EPO (5 mg/kg) mice.

**Table 3:** The effect of different doses of EPO on TNF-α and MDA levels in the liver and pancreas tissue homogenates of CP-intoxicated mice.

Groups	Liver TNF-α	Pancreas TNF-α	Liver MDA	Pancreas MDA
Control	53.6 ± 5.9	25.6 ± 5.8	2.2 ± 0.21	0.52 ± 0.15
CP-intoxicated	132.3 ± 6.8 ^a^	151.3 ± 6.9 ^a^	13.3 ± 0.57 ^a^	1.36 ± 0.06 ^a^
CP + EPO (5 mg/kg)	83.3 ± 6.7 ^ab^	78.3 ± 6.6 ^ab^	6 ± 0.78 ^ab^	0.59 ± 0.08 ^b^
CP + EPO (10 mg/kg)	56 ± 3.8 ^bc^	41.3 ± 3.9 ^ab^	3.9 ± 0.39 ^abc^	0.39 ± 0.04 ^b^

CP: cyclophosphamide, EPO: evening primrose oil, MDA: Malondialdehyde, TNF: Tumor necrosis factor. All data were expressed as mean ± SEM and analyzed using the one-way ANOVA followed by Bonferroni’s post-hoc test at *p* ˂ 0.05. ^a^ Significantly different from control mice, ^b^ Significantly different from CP-intoxicated mice, ^c^ Significantly different from CP + EPO (5 mg/kg) mice.

**Table 4:** The histopathological scoring system for (**a**) hepatic and (**b**) pancreatic tissues in CP-intoxicated mice treated with different doses of evening primrose oil.

**Groups**	**Grades**				**Mean Scoring Grades for Hepatic Histopathological Changes**
	**1**	**2**	**3**	**4**	
**Control**	7	1	0	0	1.125 ± 0.13
**CP-intoxicated**	0	1	4	3	3.25 ± 0.25 ^a^
**CP + EPO (5 mg/kg)**	0	6	1	1	2.37 ± 0.26 ^ab^
**CP + EPO (10 mg/kg)**	6	1	1	0	1.63 ± 0.42 ^b^
(**a**)					
**Groups**	**Grades**				**Mean Scoring Grades for Pancreatic Histopathological Changes**
	**1**	**2**	**3**	**4**	
**Control**	6	1	1	0	1.37 ± 0.26
**CP-intoxicated**	0	1	4	3	3.37 ± 0.18 ^a^
**CP + EPO (5 mg/kg)**	0	6	1	1	2.5 ± 0.26 ^ab^
**CP + EPO (10 mg/kg)**	6	1	1	0	1.5 ± 0.26 ^bc^
(**b**)					

CP: cyclophosphamide, EPO: evening primrose oil. All data are expressed as mean ± SEM and analyzed using the one-way ANOVA, followed by Bonferroni’s post-hoc test at *p* ˂ 0.05. ^a^ Significantly different from the control mice, ^b^ Significantly different from CP-intoxicated mice, ^c^ Significantly different from CP + EPO (5 mg/kg) mice.
